# The prognostic significance of the lactate dehydrogenase-to-albumin ratio in extensive-stage small cell lung cancer patients receiving chemo-immunotherapy: a multicenter study

**DOI:** 10.3389/fimmu.2026.1804717

**Published:** 2026-08-12

**Authors:** Xinwen Wu, Yi Li, Jie Lou, Shuai Qie, Yuxin Sun, Peng Mo, Yong Wang, Shanshan Jiang, Yuyang Wang, Shu Peng, Lian Yang, Guofeng Zhou

**Affiliations:** 1 Department of Radiology, Union Hospital, Tongji Medical College, Huazhong University of Science and Technology, Wuhan, China; 2 Hubei Provincial Clinical Research Center for Precision Radiology & Interventional Medicine, Wuhan, China; 3 Hubei Key Laboratory of Molecular Imaging, Wuhan, China; 4 Department of Radiotherapy, Affiliated Hospital of Hebei University, Baoding, China; 5 Department of Radiotherapy, 900th Hospital of Joint Logistics Support Force, Fuzhou, China; 6 Department of Radiology, Xiangyang No.1 People’s Hospital, Hubei University of Medicine, Xiangyang, China; 7 Department of Thoracic Surgery, Tongji Hospital, Tongji Medical College, Huazhong University of Science and Technology, Wuhan, China

**Keywords:** chemoimmunotherapy, immune checkpoint inhibitors, small cell lung cancer, the lactate dehydrogenase, the lactate dehydrogenase-to-albumin ratio

## Abstract

**Background:**

The lactate dehydrogenase-to-albumin ratio (LAR) has been acknowledged as an independent prognostic factor for multiple diseases. However, its prognostic significance in extensive-stage small cell lung cancer (ES-SCLC) patients treated with immune checkpoint inhibitors (ICIs) remains ambiguous. This study aims to explore the influence of LAR on the prognosis of ES-SCLC patients undergoing ICIs combined with chemotherapy.

**Methods:**

A cohort of 628 patients with ES-SCLC who received ICIs from five hospitals underwent pre-treatment blood biochemical examinations. The overall survival (OS) and progression-free survival (PFS) were analyzed via Cox regression models and Kaplan-Meier survival curves. A multivariate Cox proportional hazards model was constructed to develop a nomogram based on independent prognostic factors. The predictive performance and clinical utility of the nomogram were evaluated using the area under the time-dependent receiver operating characteristic (ROC) curve, calibration curves, and decision curve analysis (DCA).

**Results:**

LAR was identified as an independent prognostic factor influencing PFS and OS. Compared to low levels of LAR, elevated LAR is associated with shorter OS (HR = 4.97,95% CI: 3.32-7.45, P < 0.001) and PFS (HR = 3.02,95% CI: 2.17-4.21, P < 0.001). The ROC curve for OS prediction in the training set exhibited areas under the curve (AUCs) of 0.833 at 1 year and 0.874 at 2 years, whereas the internal validation set showed AUCs of 0.819 for 1- year OS and 0.891 for 2-year OS. The AUCs for 1-year PFS and 2-year PFS in the training set were 0.842 and 0.889, respectively, compared to 0.799 and 0.921 in the internal validation set. Calibration curves verified a good concordance between predicted and observed outcomes in both the training and internal validation sets. In the independent external test cohort, the calibration curve also demonstrated consistency between predicted and observed results. The AUCs for 1-year OS and 2-year OS in the external test set were 0.76 and 0.69. The AUCs for 1-year PFS and 2-year PFS in the external test set were 0.59 and 0.58.

**Conclusion:**

In patients with ES-SCLC receiving ICIs combined with chemotherapy, a high pre-treatment LAR was associated with worse clinical outcomes.

## 1 Introduction

Lung cancer stands as the primary cause of cancer-related mortalities worldwide, constituting 18% of all cancer-related fatalities (1). Small cell lung cancer (SCLC) accounts for roughly 15% of all lung cancer instances. Owing to its rapid proliferation and early metastatic propensity, 80 - 85% of patients are diagnosed with extensive-stage small cell lung cancer (ES-SCLC) upon initial examination (2, 3). SCLC is typically not suitable for surgical resection, and the current standard therapeutic approach for ES-SCLC entails a combination of chemotherapy, immunotherapy, and radiotherapy. In recent years, the advent of immune checkpoint inhibitors (ICIs), particularly antibodies targeting programmed cell death protein 1 (PD-1) or programmed death ligand 1 (PD-L1), has significantly transformed the therapeutic landscape for SCLC. Considering the demonstrated improvement in the overall survival of patients through the combination of chemotherapy and immune checkpoint inhibitors, the incorporation of PD-1/PD-L1 inhibitors into conventional chemotherapy regimens has emerged as a novel first-line standard for the treatment of SCLC (4). Large-scale clinical trials such as Impower133 (5), Astrum-005 (6), CASPIAN (7), CAPSTONE-1 (8) had demonstrated that anti-PD-1/PD-L1 combined with chemotherapy has been established as the standard treatment approach for patients diagnosed with ES-SCLC. Despite significant advances in the field of immune checkpoint therapy, only a minority of SCLC patients can benefit from ICIs, which are often associated with varying degrees of immune-related adverse events (9). Consequently, it is of great significance to explore predictive biomarkers for screening potential beneficiaries of immune checkpoint inhibitors and aiding clinicians in decision-making. PD-L1 expression (10, 11), Tumor mutational burden (TMB) (12–14), Tumor microenvironment (TME) (15, 16) may function as a valuable biomarker for forecasting the probability of SCLC response to immune checkpoint inhibitors. Nevertheless, these biomarkers are challenging to acquire, and their predictive efficacy necessitates further verification (17). The clinical demand for dependable biomarkers capable of accurately forecasting treatment efficacy and patient prognosis remains unfulfilled.

Recent research findings have indicated that nutritional status and inflammation are implicated in the initiation and progression of tumors (18, 19). Lactate dehydrogenase (LDH), an enzyme within the glycolytic pathway, participates in tumorigenesis, the immune microenvironment, and cancer progression (20, 21). Elevated LDH levels indicate inflammation, hypoxia, and necrosis (22). The high expression of LDH is also associated with the enhanced proliferation, migration and invasion of tumor cells (23). Albumin (ALB), an important plasma protein, whose level decreased indicates malnutrition or inflammation. The lactate dehydrogenase-to-albumin ratio (LAR) is a systemic inflammatory biomarker that reflects the patient’s systemic nutritional status, inflammatory state, and disease severity. Several studies have established a link between elevated LAR and rworse prognosis for various cancers (including oral cancer and colorectal cancer) (24, 25). Nevertheless, additional research is necessary to comprehensively comprehend the correlation between LAR and the clinical outcomes of ES-SCLC. In this context, investigating LAR in the setting of immune checkpoint inhibitor therapy may provide valuable prognostic insights. This research constitutes the first multicenter study in China aimed at exploring the prognostic significance of the LAR in immune checkpoint inhibitor therapy for ES-SCLC.

## 2 Methods

### 2.1 Research design and patient selection

**Figure 1:**
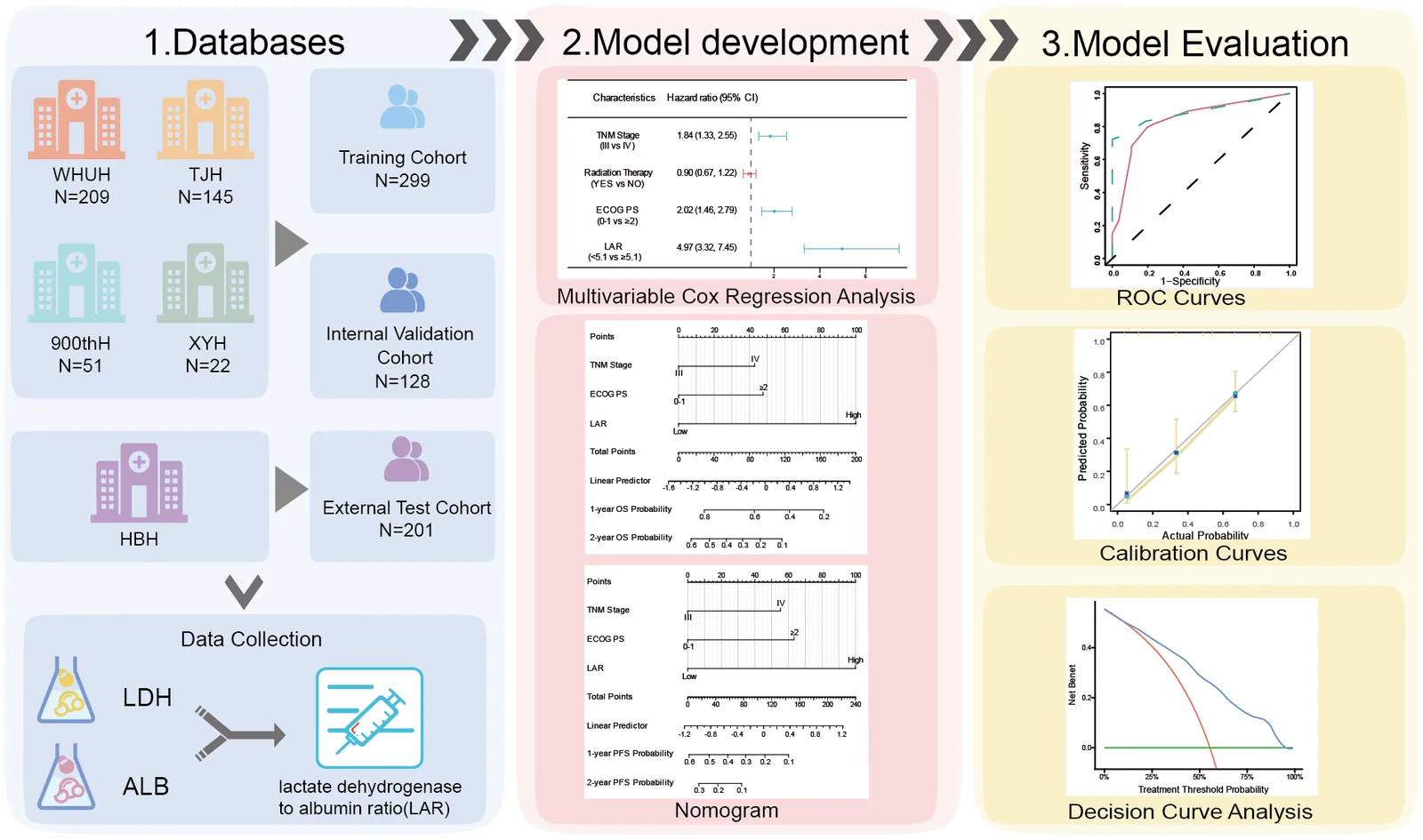
Flow diagram of enrolled participants and evaluation process. WHUH, The Union Hospital of Tongii Medical College, Huazhong University of Science and Technology: TJH, the Tongji Hospital of Tongii Medical College, Huazhong University of Science and Technology: 900thH, The 900th Hospital of the Joint Logistics Team; XYH, Xiangyang No.1 People's Hospital; HBH, Affiliated Hospital of Hebei University; LDH, Lactate dehydrogenase; ALB, Albumin; ROC, receiver operating characteristic; OS, overall survival; PFS, progression-free survival.

**Figure 1** presents the flow diagram of the present study. This retrospective study included patients diagnosed with advanced SCLC who received immune checkpoint inhibitor therapy at Wuhan Union Hospital, Wuhan Tongji Hospital, Xiangyang First People’s Hospital, The 900th Hospital of the Joint Logistics Team and Affiliated Hospital of Hebei University from January 2016 to May 2024. Baseline biochemical test results (levels of LDH and ALB) were collected from patients before ICIs treatment, and regular follow-ups were conducted six months after the start of treatment. The inclusion criteria were as follows: 1. SCLC confirmed by histological or cytological examination; 2. Combined chemotherapy with immune checkpoint inhibitors, or a combination of chemotherapy, targeted therapy, and immune checkpoint inhibitors; 3. Patients aged over 18 years; 4. Stage III or IV small cell lung cancer treated with chemoimmunotherapy, including stage III patients who were deemed unsuitable for definitive thoracic radiotherapy and therefore managed according to an extensive-stage treatment strategy; 5. Complete baseline laboratory data. The exclusion criteria were: 1. Cases without follow-up data or with missing LDH/ALB data; 2. Patients with inflammatory diseases; 3. Primary malignancies in other systems; 4. SCLC patients with a history of previous surgical treatment; 5. Patients who had received other therapies before ICIs treatment. The final data analysis included 628 eligible participants. Patients from Hebei University Affiliated Hospital constituted an independent external test cohort, whereas patients from the remaining four hospitals were randomly partitioned into a training set and an internal validation set at a ratio of 7:3.

### 2.2 Data collection

A retrospective collection was conducted on the clinical and pathological characteristics of the enrolled patients, encompassing age, gender, body mass index (BMI), Eastern Cooperative Oncology Group performance status (ECOG PS), hypertension, smoking history, lactate dehydrogenase levels, albumin, carcinoembryonic antigen (CEA), neuron-specific enolase (NSE), TNM (Tumor–Node–Metastasis) stage, lymph node metastasis, liver metastasis, brain metastasis, bone metastasis, and radiotherapy history. Simultaneously, we collected detailed treatment data for all patients, including chemotherapy, immunotherapy, and, in a subset of cases, targeted anti-angiogenic therapy. First-line chemotherapy for extensive-stage small cell lung cancer mainly consisted of EP (etoposide plus cisplatin) and EC (etoposide plus carboplatin) regimens. A proportion of patients received irinotecan or albumin paclitaxel, primarily because of intolerance to etoposide or concerns regarding renal function. In addition, a small number of patients were treated with targeted anti-angiogenic agents, mainly anlotinib and apatinib.

LAR was calculated as LDH (U/L)/serum albumin (g/L). Tumor response was evaluated via computed tomography (CT) and the Response Evaluation Criteria for Solid Tumors (RECIST) 1.1. Progression-Free Survival (PFS) was defined as the time from the start of treatment to the first occurrence of disease progression or death from any cause, whichever occurs first. Overall Survival (OS) was defined as the time from the start of treatment to death from any cause or the time of the last follow-up. The follow-up period concluded in June 2025.

### 2.3 Statistical analysis

The optimal cutoff values for LDH, ALB, and LAR were evaluated using the receiver operating characteristic (ROC) curve. The Youden index, calculated as “sensitivity + specificity – 1”, was computed, and the optimal cutoff value was selected based on the maximum Youden index. The median follow-up time was estimated using the reverse Kaplan–Meier method. Continuous variables were expressed as medians with interquartile range (IQR). Continuous variables between groups were compared using t-tests. Chi-square tests were used to analyze differences between categorical data groups. Kaplan-Meier curves and log-rank tests were employed to assess the impact of LAR on OS and PFS. Variables with p-values <0.05 in univariate Cox analysis were included in multivariate Cox analysis. Hazard ratios (HR) and 95% confidence intervals (CI) were calculated. Predictive variables with P-values <0.05 in multivariate analysis were used to construct nomogram for 1-year and 2-year OS/PFS. Subgroup analysis was performed using stratified Cox proportional hazards models. The performance of these plots was evaluated using calibration curves, Harrell’s consistency index, and time-varying ROC curves. The net benefit of these measures was assessed using decision curve analysis. P-values <0.05 were considered statistically significant. Statistical analysis was conducted using R (version 4.2.1).

## 3 Results

### 3.1 Patient characteristics

This study enrolled a total of 628 ES-SCLC patients (299 in the training set, 128 in the internal validation set and 201 in the external test set), all of whom received immune checkpoint inhibitors combined with chemotherapy. The demographic and clinical characteristics of the training and validation cohorts are presented in **Table 1**. Both cohorts showed a higher proportion of male patients and those under 65 years of age. Additionally, the proportions of patients with stage IV and those with lymph node metastasis were significantly higher in both cohorts.

**Table 1:** Comparison of clinicopathological characteristics in training, internal validation and external test sets.

Characteristic	Cohort		
	Training cohort	Internal validation cohort	External test cohort
	N = 299	N = 128	N = 201
Sex			
Male	264 (88.3%)	109 (85.2%)	141 (70.1%)
Female	35 (11.7%)	19 (14.8%)	60 (29.9%)
Age			
<65	164 (54.8%)	75 (58.6%)	125 (62.2%)
≥65	135 (45.2%)	53 (41.4%)	76 (37.8%)
Hypertension			
YES	93 (31.1%)	31 (24.2%)	86 (42.8%)
NO	206 (68.9%)	97 (75.8%)	115 (57.2%)
Smoking history			
YES	164 (54.8%)	64 (50.0%)	128 (63.7%)
NO	135 (45.2%)	64 (50.0%)	73 (36.3%)
TNM stage			
III	128 (42.8%)	51 (39.8%)	57 (28.4%)
IV	171 (57.2%)	77 (60.2%)	144 (71.6%)
Radiation therapy			
YES	169 (56.5%)	64 (50.0%)	170 (84.6%)
NO	130 (43.5%)	64 (50.0%)	31 (15.4%)
Lymph node metastasis			
YES	275 (92.0%)	120 (93.8%)	136 (67.7%)
NO	24 (8.0%)	8 (6.3%)	65 (32.3%)
Types of targeted agents			
Anlotinib	26 (8.7%)	7 (5.5%)	3 (1.5%)
Apatinib	3 (1.0%)	1 (0.8%)	2 (1.0%)
NO	270 (90.3%)	120 (93.8%)	196 (97.5%)
Chemotherapy			
EP	200 (66.9%)	87 (68.0%)	189 (94.0%)
EC	87 (29.1%)	39 (30.5%)	12 (6.0%)
Other	12 (4.0%)	2 (1.6%)	0 (0.0%)
Immunotherapy			
Pembrolizumab	8 (2.7%)	2 (1.6%)	0 (0.0%)
Sintilimab	52 (17.4%)	17 (13.3%)	131 (65.2%)
Durvalumab	71 (23.7%)	38 (29.7%)	18 (9.0%)
Atezolizumab	28 (9.4%)	15 (11.7%)	0 (0.0%)
Serplulimab	38 (12.7%)	16 (12.5%)	3 (1.5%)
Tislelizumab	66 (22.1%)	26 (20.3%)	36 (17.9%)
Camrelizumab	14 (4.7%)	6 (4.7%)	6 (3.0%)
Toripalimab	8 (2.7%)	2 (1.6%)	0 (0.0%)
Other	14 (4.7%)	6 (4.7%)	7 (3.5%)
Type of ICIs			
PD-1	193 (64.5%)	71 (55.5%)	180 (89.6%)
PD-L1	106 (35.5%)	57 (44.5%)	21 (10.4%)
Liver Metastasis			
YES	51 (17.1%)	26 (20.3%)	34 (16.9%)
NO	248 (82.9%)	102 (79.7%)	167 (83.1%)
Bone Metastasis			
YES	57 (19.1%)	28 (21.9%)	60 (29.9%)
NO	242 (80.9%)	100 (78.1%)	141 (70.1%)
Brain Metastasis			
YES	50 (16.7%)	20 (15.6%)	43 (21.4%)
NO	249 (83.3%)	108 (84.4%)	158 (78.6%)
ECOG-PS			
0-1	228 (76.3%)	99 (77.3%)	185 (92.0%)
≥2	71 (23.7%)	29 (22.7%)	16 (8.0%)
BMI			
< 25	216 (72.2%)	95 (74.2%)	108 (53.7%)
≥25	83 (27.8%)	33 (25.8%)	93 (46.3%)
LAR-continue			
Mean ± SD	7.20 ± 8.40	8.12 ± 9.06	5.39 ± 1.94
LAR			
Low	111 (37.1%)	35 (27.3%)	104 (51.7%)
High	188 (62.9%)	93 (72.7%)	97 (48.3%)
CEA			
Mean ± SD	35 ± 212	27 ± 85	NA
NSE			
Mean ± SD	62 ± 79	56 ± 66	NA
The median follow-up time for OS (days, 95%CI)	902 (777 – 1081)	993 (816 – 1055)	1506 (1345 – 1548)
The median follow-up time for PFS (days, 95%CI)	841 (708 – 1094)	Not Reached	1506 (1204 – 1673)
OS Event	189	93	107
PFS Event	237	118	175

Note. TNM, tumor–node–metastasis; EP, Etoposide+Cisplatin; EC, Etoposide+Carboplatin; ICIs, immune checkpoint inhibitors; PD-1, programmed cell death protein 1; PD-L1, programmed cell death ligand 1; ECOG PS, Eastern Cooperative Oncology Group Performance Status; LAR, lactate dehydrogenase to albumin ratio; BMI, Body Mass Index; CEA, carcinoembryonic antigen; NSE, neuron-specific enolase.

### 3.2 The optimal cutoff value of LAR and its correlation with clinical pathological features

During the discovery phase, the optimal threshold for LAR was determined as 5.1 based on the maximum Youden index under the ROC curve of the patients’ survival outcome in the training cohort (**Supplementary Figure 1**), with a sensitivity of 0.645 and specificity of 0.809 (**Supplementary Table 1**). Based on the optimal threshold of 5.1, patients were categorized into two groups: the high-LAR group and the low-LAR group. The high-LAR group comprised 188 patients (62.9%), while the low-LAR group included 111 patients (37.1%). In the training set, both the high-LAR and low-LAR groups demonstrated a higher proportion of male patients, those aged <65 years, and those with lymph node metastasis (**Supplementary Table 2**). Similar patient characteristics were observed within the internal validation set and external test set (**Supplementary Tables 3**, **4**).

### 3.3 Tumor response

When evaluating treatment efficacy in patients with high and low LAR levels, we observed an objective response rate (ORR) of 44.1% in the low LAR group and 39.4% in the high LAR group in the training set, showing no statistically significant difference (P = 0.425). Similar results were observed in the internal validation set (31.4% vs 31.2%, P = 0.997) (**Supplementary Table 4**). The training set demonstrated that low LAR was associated with higher disease control rate (DCR) (85.6% vs 73.4%, P = 0.008), but this trend was not observed in the internal validation set (77.1% vs 69.9%, P = 0.397) (**Supplementary Table 4**).

### 3.4 Survival analysis

**Figure 2:**
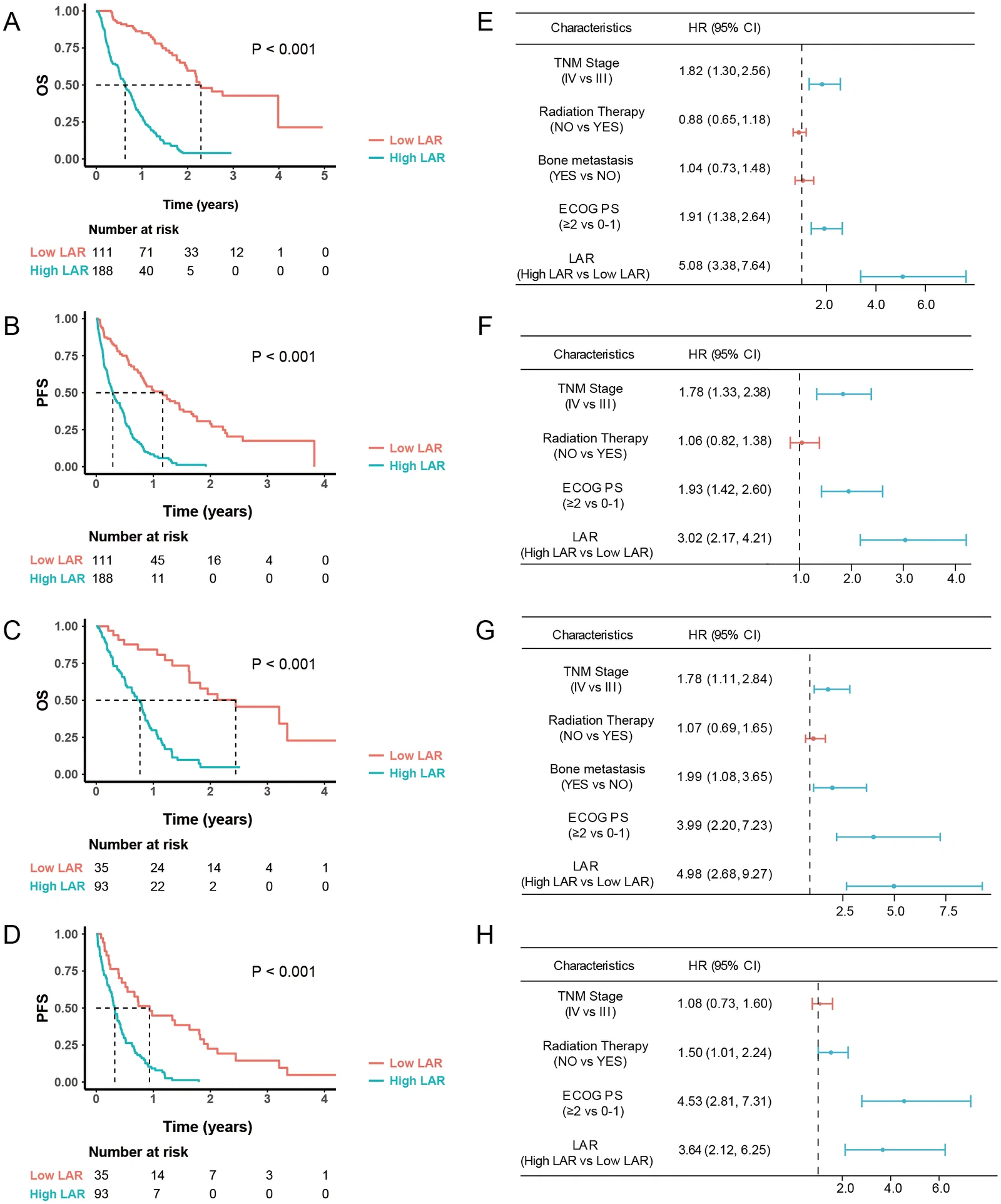
Prognostic implications of the LAR. Kaplan-Meier curves of OS **(A)** and PFS **(B)** for patients in the low- and high-LAR groups in the training set. Kaplan-Meier curves of OS **(C)** and PFS **(D)** for patients in the low- and high-LAR groups in the validation set. Forest plot of multivariable Cox regression analysis of OS **(E)** and PFS **(F)** in the training set. Forest plot of multivariable Cox regression analysis of OS **(G)** and PFS **(H)** in the validation set. OS, overall survival; PFS, progression-free survival; LAR, lactate dehydrogenase to albumin ratio: HR. Hazard ratio: TNM, tumor-node-metastasis; ECOG PS. Eastern Cooperative Oncology Group Performance Status.

The Kaplan-Meier survival curves for OS (**Figure 2A**) and PFS (**Figure 2B**) in patients with low-level LAR versus high-level LAR, as shown by the log-rank test in the training set, demonstrated that patients with low-level LAR had significantly longer PFS and OS than those with high-level LAR (OS, P < 0.001; PFS, P < 0.001). During the verification phase, this finding was also confirmed in the internal validation cohort (OS, P < 0.001; PFS, P < 0.001) (**Figures 2C, D**). This substantiates the prognostic significance of LAR.

Furthermore, this study conducted subgroup survival analysis based on TNM staging. The results demonstrated that, regardless of stage III (**Supplementary Figures 2A–D**) or IV (**Supplementary Figures 2E–H**), grouping based on the LAR cutoff value exhibited favorable stratification effects, which were statistically significant for both OS and PFS.

### 3.5 COX regression analysis

Univariate Cox analysis of the training set identified TNM stage, prior radiotherapy, bone metastasis, ECOG PS, and LAR as potential predictors of OS. For PFS, the potential predictors included TNM stage, prior radiotherapy, ECOG PS, and LAR (**Table 2**). These factors were incorporated into multivariate Cox regression analysis. The results showed a higher TNM staging (OS: HR = 1.84; 95% CI: 1.33-2.55, P < 0.001; PFS: HR = 1.78; 95% CI: 1.33-2.38, P < 0.001), higher ECOG PS (OS: HR = 2.02; 95% CI: 1.46-2.79, P < 0.001; PFS: HR = 1.93; 95% CI: 1.42-2.60, P < 0.001), higher LAR (OS: HR = 4.97,95% CI: 3.32-7.45, P < 0.001; PFS: HR = 3.02,95% CI: 2.17-4.21, P < 0.001) significantly associated with shorter OS (**Figure 2E**) and PFS (**Figure 2F**). In the internal validation cohort, both univariate and multivariate analysis revealed significant correlations between the higher LAR and short OS (**Figure 2G**) as well as PFS (**Figure 2H**) (OS: HR = 5.05; 95% CI: 2.70-9.46; P < 0.001; PFS: HR = 3.64; 95% CI: 2.12-6.25; P< 0.001). Furthermore, risk factor maps based on Cox analysis were constructed in both cohorts, demonstrating a strong correlation between high-risk status and high LAR in both the training set (**Supplementary Figure 3**) and internal validation set (**Supplementary Figure 4**). To further evaluate the robustness of LAR, we also analyzed LAR as a continuous variable in Cox regression models. Continuous LAR remained significantly associated with OS and PFS in the training and internal validation cohorts. In the external validation cohort, LAR was significantly associated with OS but not with PFS. Nevertheless, the HR for PFS was greater than 1 (HR = 1.056), indicating a directionally consistent but non-significant trend (**Supplementary Tables 8**, **9**).

**Table 2:** Results of univariate survival analysis in training, internal validation and external test sets.

Univariate COX regression analysis for OS						
Characteristics	Training set		Internal validation set		External test set	
	Hazard ratio (95% CI)	P value	Hazard ratio (95% CI)	P value	Hazard ratio (95% CI)	P value
Sex (Female vs Male)	1.006 (0.649 - 1.560)	0.978	0.986 (0.557 - 1.747)	0.962	0.642 (0.412 - 0.999)	**0.050**
Age (≥65 vs <65)	1.180 (0.886 - 1.573)	0.257	0.996 (0.653 - 1.519)	0.984	1.091 (0.739 - 1.609)	0.662
Hypertension (NO vs YES)	1.067 (0.784 - 1.452)	0.679	1.278 (0.782 - 2.088)	0.328	1.054 (0.715 - 1.553)	0.790
Smoking History (NO vs YES)	1.002 (0.753 - 1.334)	0.989	0.918 (0.609 - 1.386)	0.685	0.729 (0.485 - 1.095)	0.128
TNM Stage (IV vs III)	2.699 (1.973 - 3.691)	**< 0.001**	1.735 (1.129 - 2.667)	**0.012**	0.929 (0.611 - 1.412)	0.730
Radiation Therapy (NO vs YES)	1.451 (1.086 - 1.938)	**0.012**	1.471 (0.964 - 2.244)	0.074	1.628 (1.016 - 2.608)	**0.043**
Lymph Node Metastasis (NO vs YES)	0.617 (0.344 - 1.109)	0.106	0.380 (0.119 - 1.206)	0.100	0.900 (0.596 - 1.359)	0.617
Types of targeted agents (Apatinib vs Anlotinib)	1.087 (0.325 - 3.635)	0.892	0.836 (0.100 - 6.975)	0.868	0.591 (0.054 - 6.528)	0.668
Types of targeted agents (NO vs Anlotinib)	1.199 (0.766 - 1.877)	0.426	1.552 (0.675 - 3.569)	0.301	1.068 (0.263 - 4.332)	0.927
Chemotherapy (EC vs EP)	0.751 (0.540 - 1.044)	0.088	0.766 (0.481 - 1.218)	0.260	0.868 (0.380 - 1.983)	0.738
Chemotherapy (Other vs EP)	1.305 (0.684 - 2.488)	0.420	1.650 (0.398 - 6.830)	0.490	NA	NA
Type of ICIs (PD-L1 vs PD-1)	0.822 (0.613 - 1.102)	0.190	0.668 (0.440 - 1.014)	0.058	0.935 (0.500 - 1.749)	0.833
Liver Metastasis (NO vs YES)	0.980 (0.684 - 1.405)	0.912	0.937 (0.550 - 1.597)	0.811	1.241 (0.738 - 2.089)	0.416
Bone Metastasis (NO vs YES)	0.671 (0.481 - 0.937)	**0.019**	1.011 (0.609 - 1.678)	0.967	0.981 (0.647 - 1.487)	0.927
Brain Metastasis (NO vs YES)	1.008 (0.691 - 1.469)	0.968	1.807 (0.982 - 3.325)	0.057	0.930 (0.585 - 1.479)	0.760
ECOG PS (≥2 vs 0-1)	2.296 (1.671 - 3.154)	**< 0.001**	3.534 (2.154 - 5.799)	**< 0.001**	1.329 (0.670 - 2.634)	0.416
BMI (≥25 vs <25)	1.318 (0.959 - 1.811)	0.088	0.809 (0.507 - 1.291)	0.374	0.722 (0.490 - 1.062)	0.098
LAR (High vs Low)	6.269 (4.294 - 9.151)	**< 0.001**	5.116 (2.833 - 9.240)	**< 0.001**	2.870 (1.923 - 4.285)	**< 0.001**
CEA	1.000 (1.000 - 1.001)	0.246	1.002 (1.000 - 1.004)	0.102	NA	NA
NSE	1.001 (0.999 - 1.003)	0.333	1.003 (1.000 - 1.006)	0.061	NA	NA
Univariate COX regression analysis for PFS						
Sex (Female vs Male)	1.375 (0.937 - 2.020)	0.104	0.986 (0.557 - 1.747)	0.962	0.921 (0.660 - 1.286)	0.629
Age (≥65 vs <65)	0.828 (0.640 - 1.072)	0.152	0.996 (0.653 - 1.519)	0.984	1.012 (0.747 - 1.371)	0.937
Hypertension (NO vs YES)	1.035 (0.787 - 1.362)	0.804	1.278 (0.782 - 2.088)	0.328	1.091 (0.807 - 1.476)	0.572
Smoking History (NO vs YES)	0.925 (0.715 - 1.197)	0.553	0.918 (0.609 - 1.386)	0.685	1.034 (0.757 - 1.413)	0.832
TNM Stage (IV vs III)	2.310 (1.755 - 3.040)	<**0.001**	1.735 (1.129 - 2.667)	**0.012**	0.843 (0.606 - 1.171)	0.308
Radiation Therapy (NO vs YES)	1.331 (1.029 - 1.723)	**0.029**	1.471 (0.964 - 2.244)	0.074	1.029 (0.686 - 1.543)	0.891
Lymph Node Metastasis ((NO vs YES)	0.757 (0.455 - 1.260)	0.285	0.380 (0.119 - 1.206)	0.100	0.903 (0.656 - 1.243)	0.532
Types of targeted agents (Apatinib vs Anlotinib)	1.075 (0.323 - 3.577)	0.906	0.836 (0.100 - 6.975)	0.868	3.229 (0.532 - 19.613)	0.203
Types of targeted agents (NO vs Anlotinib)	1.027 (0.673 - 1.569)	0.900	1.552 (0.675 - 3.569)	0.301	1.259 (0.401 - 3.954)	0.693
Chemotherapy (EC vs EP)	0.837 (0.630 - 1.113)	0.222	0.766 (0.481 - 1.218)	0.260	0.995 (0.539 - 1.837)	0.986
Chemotherapy (Other vs EP)	1.130 (0.594 - 2.149)	0.709	1.650 (0.398 - 6.830)	0.490	NA	NA
Type of ICIs (PD-L1 vs PD-1)	0.978 (0.753 - 1.270)	0.868	0.668 (0.440 - 1.014)	0.058	0.707 (0.422 - 1.185)	0.188
Liver Metastasis (NO vs YES)	0.938 (0.676 - 1.303)	0.703	0.937 (0.550 - 1.597)	0.811	1.223 (0.810 - 1.847)	0.337
Bone Metastasis (NO vs YES)	0.841 (0.611 - 1.159)	0.290	1.011 (0.609 - 1.678)	0.967	1.140 (0.818 - 1.590)	0.439
Brain Metastasis (NO vs YES)	0.919 (0.652 - 1.296)	0.631	1.807 (0.982 - 3.325)	0.057	0.766 (0.534 - 1.098)	0.147
ECOG PS (≥2 vs 0-1)	2.224 (1.659 - 2.981)	<**0.001**	3.534 (2.154 - 5.799)	**< 0.001**	1.688 (1.007 - 2.831)	**0.047**
BMI (≥25 vs <25)	1.202 (0.904 - 1.598)	0.205	0.809 (0.507 - 1.291)	0.374	1.253 (0.930 - 1.688)	0.137
LAR (High vs Low)	3.981 (2.903 - 5.457)	<**0.001**	5.116 (2.833 - 9.240)	**< 0.001**	1.550 (1.147 - 2.093)	**0.004**
CEA	1.000 (1.000 - 1.001)	0.758	1.002 (1.000 - 1.004)	0.102	NA	NA
NSE	1.001 (0.999 - 1.002)	0.456	1.003 (1.000 - 1.006)	0.061	NA	NA

TNM, tumor–node–metastasis; EC, Etoposide+Carboplatin; EP, Etoposide+Cisplatin; ICIs, immune checkpoint inhibitors; PD-1, programmed cell death protein 1; PD-L1, programmed cell death ligand 1; ECOG PS, Eastern Cooperative Oncology Group Performance Status; LAR, lactate dehydrogenase to albumin ratio; BMI, body mass index; CEA, carcinoembryonic antigen; NSE, neuron-specific enolase; OS, overall survival; PFS, progression-free survival; CI, Confidence Interval.

Bold values represent statistically significant results with P < 0.05.

### 3.6 Subgroup analysis

Subsequent subgroup analysis revealed that within the training set, patients in the High LAR group exhibited significantly shorter OS (**Supplementary Figure 5**) compared to the Low LAR group (HR = 6.23, 95% CI: 4.27–9.08, P<0.001), demonstrating a consistently elevated mortality risk across all subgroups. Furthermore, PFS (**Supplementary Figure 6**) was significantly reduced in the High LAR group, with significant interactions observed in the sex (P for interaction=0.021) and smoking history (P for interaction=0.021) subgroups. In the internal validation set, High LAR was also associated with a higher risk of death (HR = 5.04, 95% CI: 2.79–9.11, P<0.001) and showed significant interaction with smoking history and TNM staging subgroups (**Supplementary Figure 7**). High LAR was also linked to an increased risk of disease progression (HR = 3.66, 95% CI: 2.19–6.12, P<0.001), with an interaction between gender and type of ICIs (**Supplementary Figure 8**).

### 3.7 Construction and verification of nomogram

**Figure 3:**
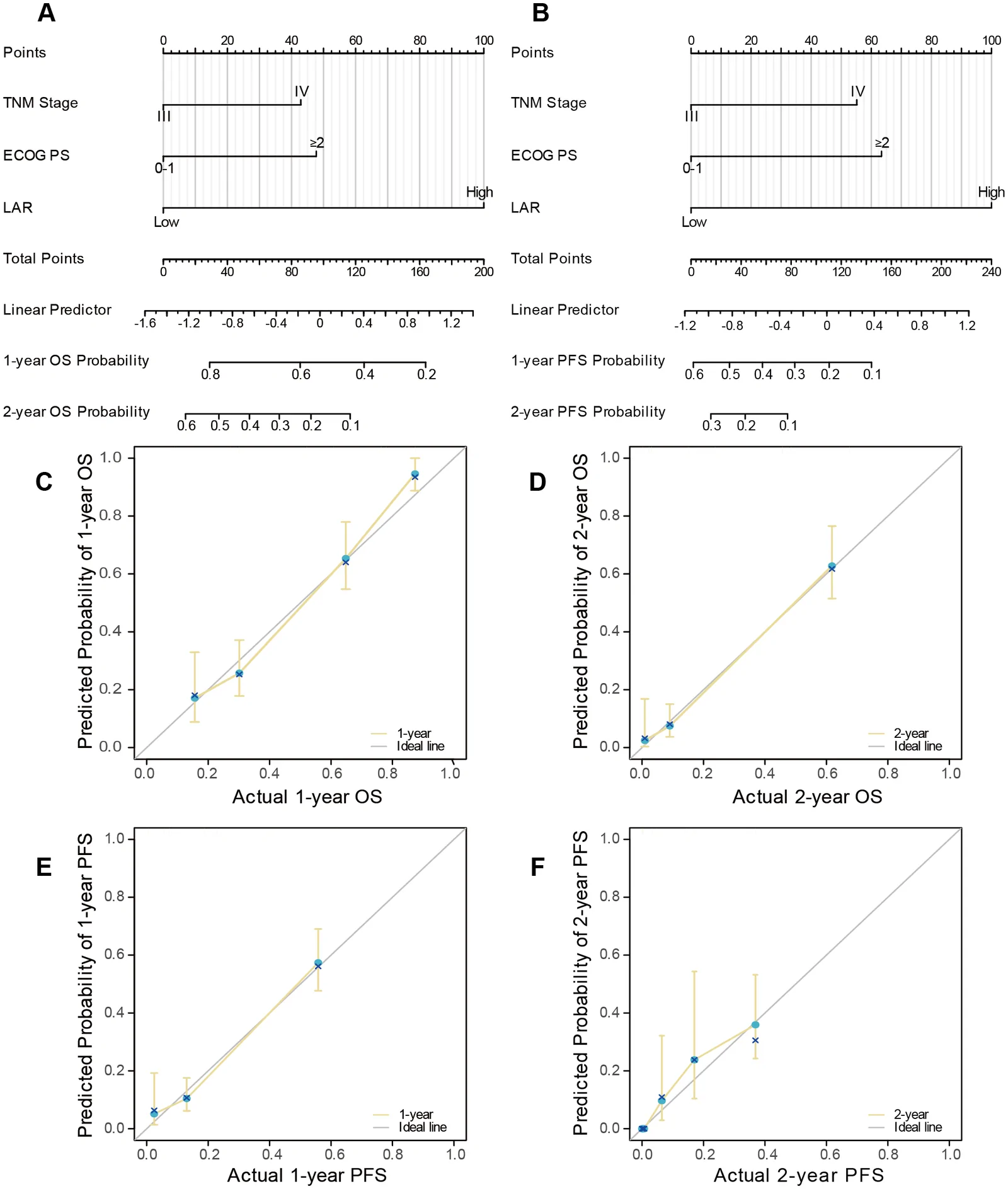
Construction and validation of the nomograms. Nomogram incorporating the LAR and other clinicopathological parameters for OS **(A)** and PFS **(B)** prediction in the training cohort. The calibration curves of the nemogram between predicted and observed 1 and 2-year OS of patients in the training set **(C, D)**. The calibration curves of the nomogram between predicted and observed 1- and 2-year PFS of patients in the training set **(E, F)**. TNM, tumor-node-metastasis; LAR, Ictate dehydrogenase to albumin ratio; ECOG PS, Eastern Cooperative Oncology Group Performance Status; OS, overall survival; PFS, progression-free survival.

**Figure 4:**
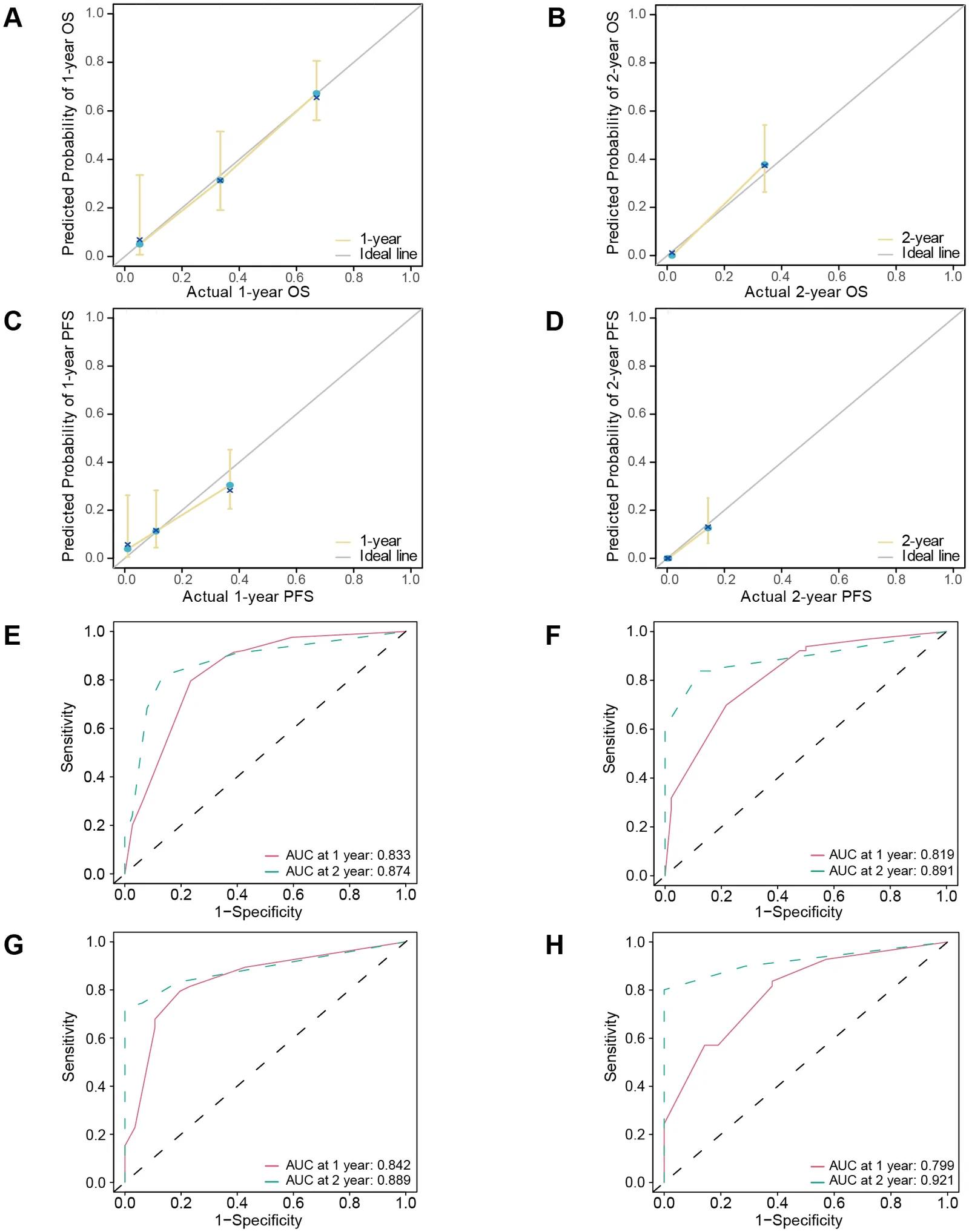
Validation of the nomograms. The calibration curves of the nomograms between predicted and observed 1- and 2-year OS **(A, B)** and PFS **(C, D)** of patients in the validation set. Time-dependent ROC curves of the nomogram for the prediction of OS in the training set **(E)** and the validation set **(F)**. Time-dependent ROC curves of the nomogram for the prediction of PFS in the training set **(G)** and the validation set **(H)**. OS, overall survival: PFS, progression-free survival: AUC, the area under the curve; ROC, receiver operating characteristic.

**Figure 5:**
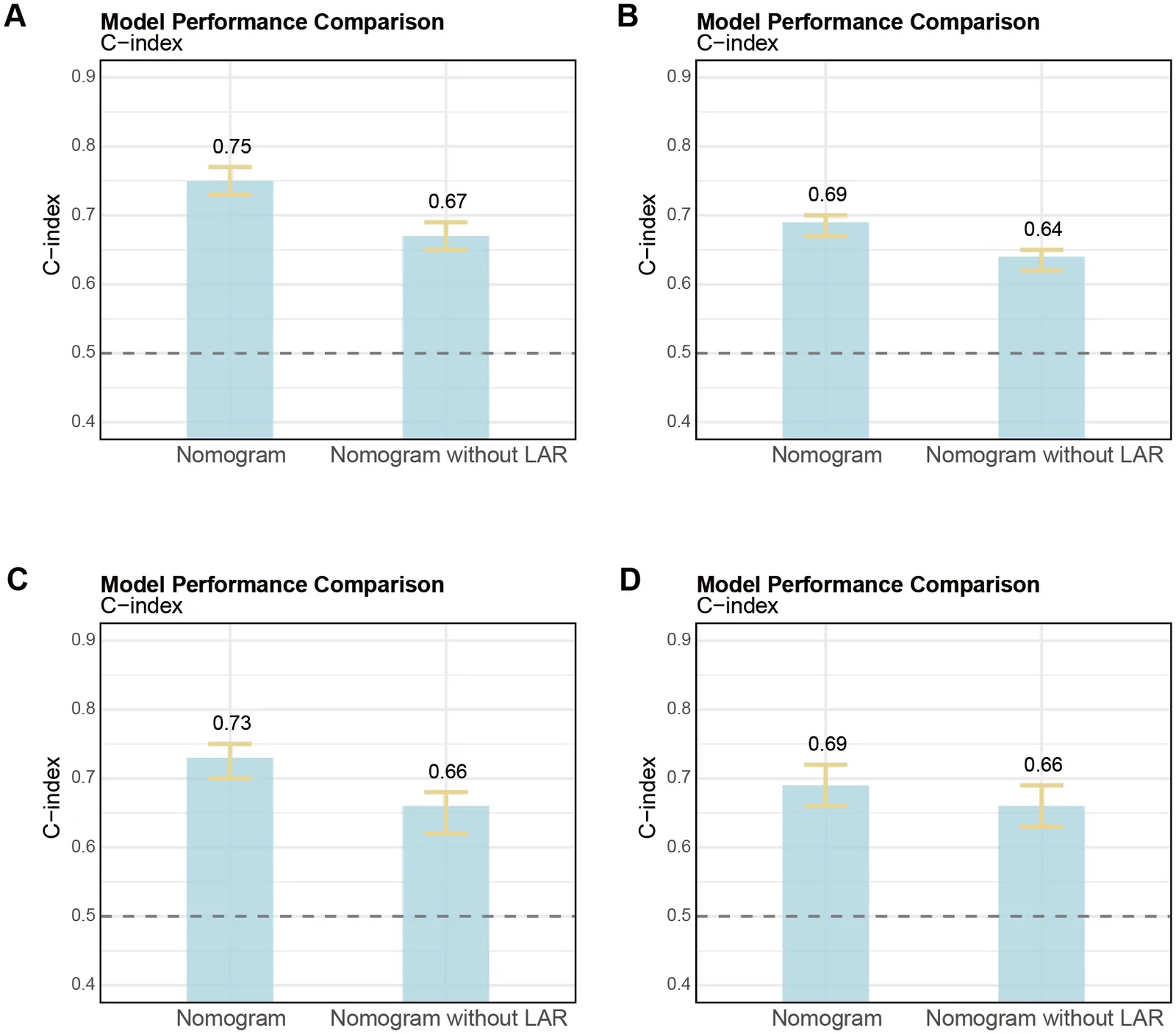
Model performance comparison. C-Index of the Nomogram and the Nomogram without LAR for the Prediction of OS **(A)** and PFS **(B)** in the training set. C- Index of the Nomogram and the Nomogram without LAR for the Prediction of OS **(C)** and PFS **(D)** in the validation set. LAR, lactate dehydrogenase to albumin ratio; OS, overall survival; PFS, progression-free survival.

According to the results of multivariate analysis, TNM stage, ECOG PS, and LAR were included in the nomogram model for 1-year and 2-year OS (**Figure 3A**) and PFS (**Figure 3B**). Based on the multivariate Cox regression model constructed from the final inclusion column plot, proportional hazards hypothesis tests were conducted for the training set. The results showed that the global tests for OS and PFS models in training cohorts yielded P> 0.05, indicating that the models satisfied the proportional hazards assumption (**Supplementary Table 5**). The AUCs of the 1-year and 2-year OS curves were 0.833 (95% CI: 0.782-0.884) and 0.874 (95% CI: 0.814-0.934), respectively (**Figure 4E**). Meanwhile, the AUCs of the 1-year and 2-year PFS curves were 0.842 (95% CI: 0.785-0.898) and 0.889 (95% CI: 0.847-0.930), respectively (**Figure 4G**). The calibration curve showed that the observed survival results were in good agreement with the model predictions (**Figures 3C–F**). Meanwhile, the clinical application of the DCA evaluation line chart was conducted by quantifying the clinical net benefit (**Supplementary Figure 9**). During the validation phase, these results were confirmed in the internal validation set. The ROC curves demonstrated that the AUCs for 1-year and 2-year OS were 0.819 (95% CI: 0.744-0.895) and 0.891(95% CI: 0.829-0.953), respectively (**Figure 4F**), while the AUCs for 1-year and 2-year PFS were 0.799 (95% CI: 0.703-0.95) and 0.921 (95% CI: 0.872-0.971) (**Figure 4H**). The calibration curves also performed well (**Figures 4A–D**). Finally, we compared this model with a counterpart excluding LAR (incorporating only TNM Stage and ECOG PS). The results demonstrate that the model incorporating LAR achieved superior discriminative performance, as evidenced by higher C-index values for both OS and PFS within the training set (OS: 0.75 vs 0.67; PFS: 0.69 vs 0.64) (**Figures 5A,B**). Correspondingly, within the internal validation set, the model incorporating LAR also demonstrated higher C-index values (OS: 0.73 vs 0.66; PFS: 0.69 vs 0.66) (**Figures 5C, D**) (**Supplementary Table 6**). In addition, we have developed two online dynamic nomograms for clinicians to use (**Supplementary Table 12**).

### 3.8 Independent external verification

**Figure 6:**
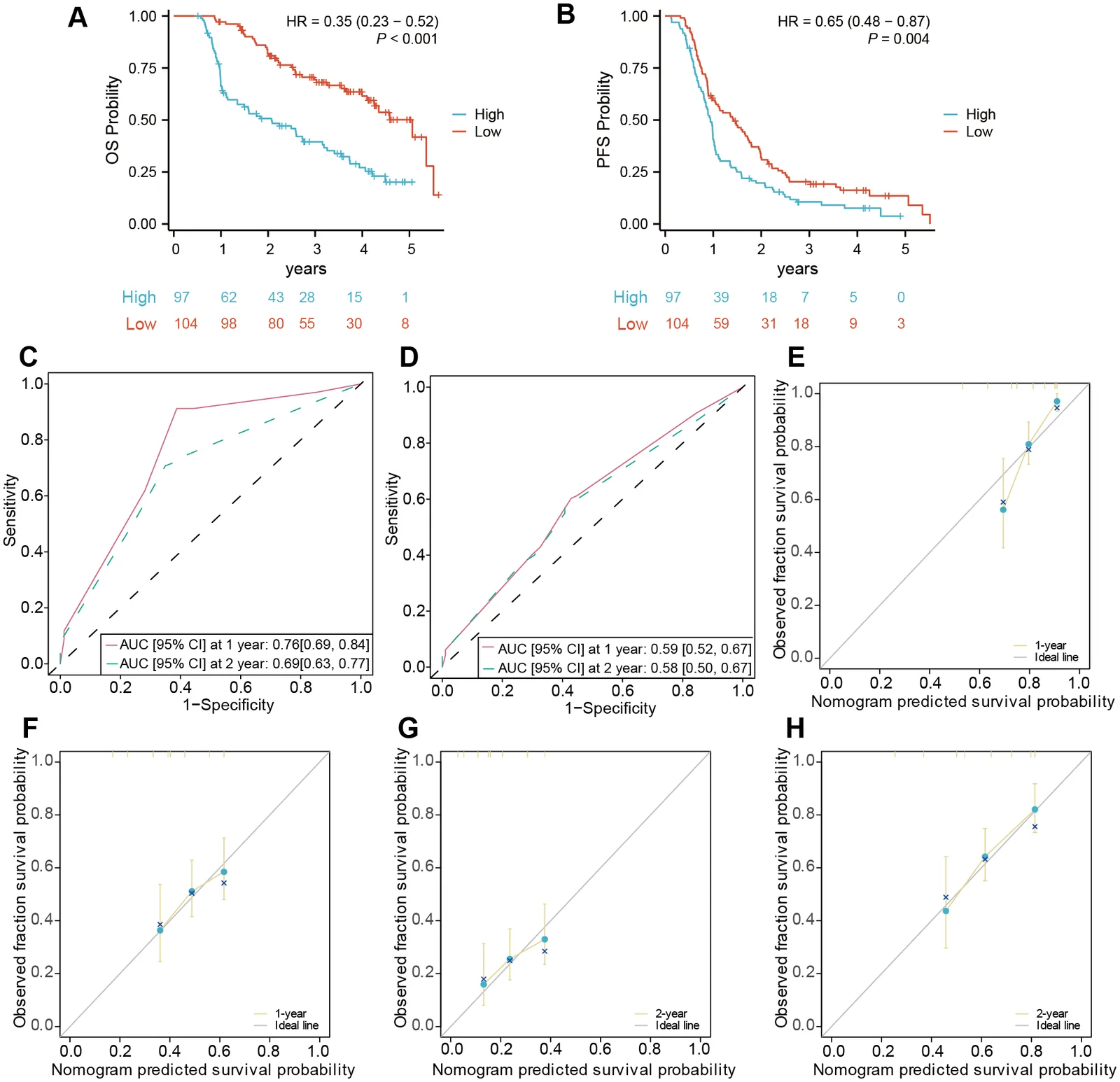
The Model Evaluation in the external test set. The Kaplan-Meier curves for OS **(A)** and PFS **(B)** of patients in the external test set. Time-dependent ROC curves of the nomogram for the prediction of OS **(C)** and PFS **(D)** in the external test set. The calibration curves of the nomogram between predicted and observed 1 and 2-year OS **(E, F)** of patients in the external test set. The calibration curves of the nomogram between predicted and observed 1-and 2-year PFS **(G, H)** of patients in the external test set.

To further validate the performance of the constructed model, we introduced an independent external validation cohort (N = 201). We first conducted survival analysis, and the Kaplan-Meier survival curves demonstrated that a high level of LAR was associated with shorter OS (P<0.001) and PFS (P = 0.004) (**Figures 6A, B**). Moreover, subgroup analyses were carried out. The results indicated that in the external test set, the OS of patients in the high LAR group was notably shorter than that in the low LAR group (HR = 2.87, 95% CI: 1.92–4.29, P < 0.001) (**Supplementary Figure 9**). A significant interaction was detected in the lymph node metastasis subgroup (P for interaction = 0.023). Additionally, the PFS in the high LAR group was significantly diminished (HR = 1.55, 95% CI: 1.50–2.09, P = 0.004) (**Supplementary Figure 10**). Subsequently, we plotted time-dependent ROC curves based on the nomogram constructed from the training set. The ROC curves revealed that the AUCs for 1-year and 2-year OS were 0.79 and 0.69 (**Figure 6C**), respectively, while the AUCs for 1-year and 2-year PFS were 0.59 and 0.58 (**Figure 6D**). The calibration curve showed good agreement between the predicted and actual outcomes (**Figures 6E–H**), and the DCA demonstrated a net benefit of the model within a certain range (**Supplementary Figure 11I–L**). we compared this model with a counterpart excluding LAR. We also compared this model with the model excluding the LAR. The model integrating LAR exhibited better discriminative performance, which was manifested by the higher C - index values for OS (0.65 vs 0.49) and PFS (0.57 vs 0.50) in the external test set (**Supplementary Table 7**).

## 4 Discussion

In this study, we propose the utilization of the LAR biomarker for predicting survival outcomes in patients with ES-SCLC, thereby facilitating clinical decision-making. We enrolled patients from multiple centers in China. Our findings demonstrate that an elevated LAR level constitutes an independent prognostic determinant for both OS and PFS. Furthermore, we developed a nomogram incorporating LAR and validated its predictive accuracy and clinical applicability.

LAR represents a robust prognostic factor independent of TNM stage and ECOG-PS, retaining its stratification capacity across diverse stage subgroups. The constructed nomogram integrates LAR, TNM stage, and ECOG PS. It demonstrates high AUCs and exhibits good calibration curves in predicting 1- and 2-year survival rates. Clinical net benefit was confirmed by DCA. Moreover, the inclusion of LAR significantly enhanced the model’s discriminative ability (C-index), substantiating its incremental prognostic value.

LDH is an enzyme implicated in cellular metabolism. Throughout the process of cancer development, tumor cells modify metabolic pathways to facilitate sustained proliferation, consequently fostering tumor cell survival and invasion. This particular phenomenon is referred to as the “Warburg effect” (26). Lactic acid serves as the principal product of this metabolic pathway. It not only modifies the tumor microenvironment but also facilitates the induction of immune-suppressive cells by tumor cells, both of which potentially impact the efficacy of immune checkpoint therapy (27, 28). Multiple studies have demonstrated that high LDH is significantly associated with worse prognosis in lymphoma, renal cell carcinoma, and nasopharyngeal carcinoma (29). It also serves as an independent prognostic factor for melanoma (30).

As an important plasma protein, albumin provides a convenient method for evaluating the function of visceral proteins and reflects the nutritional status of patients (31). Especially during the disease progression stage, malnutrition and inflammation can deplete protein reserves and inhibit protein synthesis, leading to hypoproteinemia (32, 33). Low serum albumin has been proved to be an independent prognostic indicator of many cancers such as lung cancer, pancreatic cancer and gastric cancer (32, 34).

Therefore, LAR integrates information from both LDH and ALB, providing a more reliable prognostic assessment of the patient’s inflammatory, immune, and nutritional status compared to LDH and ALB alone. LAR has been validated in multiple studies for predicting the prognosis of patients with various cancers. Peng et al. conducted a study involving 1,661 nasopharyngeal carcinoma (NPC) patients, confirming that LAR is an independent prognostic factor for NPC (35). The cutoff value of LAR varies among different cancers, which may be related to tumor type, progression rate, invasiveness, malignancy, and study population selection (36).

In this study, no statistically significant difference was observed in the ORR between the training cohort and the internal validation cohort. Additionally, the results for the DCR were inconsistent. These findings suggest that, within the current dataset, the pre-treatment LAR may not be a reliable indicator for distinguishing short-term treatment responses; however, this biomarker could serve as a significant indicator reflecting patient prognosis. ECOG PS was a significant prognostic factor for patients with ES-SCLC, and history of radiotherapy may be associated with patient survival prognosis. But in this study, only the presence of radiotherapy history could be confirmed, along with detailed descriptions of the patients’ intent and timing for radiotherapy. Although other studies have similarly demonstrated that radiotherapy improves overall survival in patients, further research is still required in the future (37, 38). We also observed that non-smokers may have a higher risk of disease progression compared to smokers, which may be attributed to the fact that SCLC in non-smokers tends to include more elderly patients, higher ECOG PS, and more advanced-stage cases, thereby leading to poorer disease outcomes (39). Furthermore, a study found that smoking induces more DNA mutations, leading to higher TMB (40). A higher TMB is associated with an increased likelihood of response to ICIs, potentially conferring greater therapeutic benefit from immunotherapy among smokers (41). A study revealed that patients with SCLC exhibiting high TMB demonstrated significantly elevated ORR and prolonged median overall survival compared with those possessing medium or low TMB levels (42).

When LAR was analyzed as a continuous variable, its prognostic association with OS remained consistent across the training, internal validation, and external validation cohorts. However, in the external validation cohort, the association between continuous LAR and PFS did not reach statistical significance, although the direction of the effect remained consistent. This discrepancy may be partly attributable to inter-cohort heterogeneity and the retrospective multicenter design. Compared with OS, PFS is more susceptible to variations in imaging follow-up intervals, response assessment criteria, treatment strategies, and documentation of disease progression. Moreover, as discussed above, LAR functions as a biomarker reflecting inflammatory, immune, and nutritional status, thereby capturing the overall clinical condition of patients. Therefore, the prognostic value of LAR may be more robust for OS than for PFS. Our study demonstrates several advantages: Firstly, we are the first to report the prognosis of patients with ES-SCLC in an Asian population receiving ICIs combined with chemotherapy; secondly, our study population was sourced from multiple regions and hospitals, enhancing the credibility of the results; 3. Subgroup analysis were conducted to confirm the robustness of the findings.

Several limitations must be acknowledged. Firstly, due to the retrospective multicenter design, the detailed data related to radiotherapy are not standardized. Consequently, radiotherapy was analyzed solely as a binary historical variable, making it impossible to accurately assess the intent-to-treat and event outcomes. Therefore, the association between radiotherapy and survival outcomes should be interpreted with caution. Secondly, data were missing regarding PD-L1 expression levels and TMB. Thirdly, this was a retrospective study, making selection bias and information bias inevitable. Although we attempted to minimize bias through predefined inclusion and exclusion criteria, unmeasured confounding factors may still have influenced the results. Fourthly, the cutoff value determined by ROC/Youden analysis demonstrated consistent prognostic stratification ability across the training, internal validation, and external validation cohorts. supporting its clinical applicability. Nevertheless, because this cutoff was derived using a data-driven approach, its generalizability should be further validated in prospective studies.

## 5 Conclusion

In summary, the present study verifies that the LAR prior to treatment serves as an independent prognostic biomarker in ES-SCLC treated with ICIs and chemotherapy. In comparison to models that do not include LAR, the integration of this cost - effective and reliable biomarker into the prognostic nomogram for ES-SCLC can yield additional net benefits for OS and PFS. Additional research is necessary to corroborate the findings of this study.

## Data Availability

The raw data supporting the conclusions of this article will be made available by the authors, without undue reservation.
