# Supplementary material for: The prognostic significance of the lactate dehydrogenase-to-albumin ratio in extensive-stage small cell lung cancer patients receiving chemo-immunotherapy: a multicenter study
Source: Front Immunol. 2026 Aug 12;17:1804717. doi: 10.3389/fimmu.2026.1804717 (PMC13506767; doi:10.3389/fimmu.2026.1804717)
Supplement: Supplementary file 1 [file Table1.docx]

**Supplementary Material**

**Content**

[**Supplementary Material**	1](#_Toc228212242)

[Supplementary Tables	2](#_Toc228212243)

[Table S1 Results from receiver operating characteristic analysis.	2](#_Toc228212244)

[Table S2 Correlations between LAR and clinicopathological characteristics of patients in training set.	3](#_Toc228212245)

[Table S3 Correlations between LAR and clinicopathological characteristics of patients in internal validation set.	5](#_Toc228212246)

[Table S4 Correlations between LAR and clinicopathological characteristics of patients in external test set.	7](#_Toc228212247)

[Table S5 Summary of confirmed response assessed by RECIST Version 1.1 in all patients.	9](#_Toc228212248)

[Table S6 Proportional hazards assumption test for the multivariable Cox regression model of OS and PFS in the training set	10](#_Toc228212249)

[Table S7 Comparison of C-index values for different prognostic models	11](#_Toc228212250)

[Table S8 Results of univariate survival analysis in training, internal validation and external test sets.	12](#_Toc228212251)

[Table S9 Results of multivariable Cox regression analysis in training, internal validation and external test sets.	14](#_Toc228212252)

[Supplementary Figures	16](#_Toc228212253)

[Figure S1 The ROC curves of training set	16](#_Toc228212254)

[Figure S2 Kaplan–Meier curves for OS and PFS stratified by the LAR in the training set and internal validation set.	17](#_Toc228212255)

[Figure S3 Risk Factor Map of training set.	18](#_Toc228212256)

[Figure S4 Risk Factor Map of validation set.	19](#_Toc228212257)

[Figure S5 Subgroup analysis for OS (Low vs High LAR) in the training set.	20](#_Toc228212258)

[Figure S6 Subgroup analysis for PFS (Low vs High LAR) in the training set.	21](#_Toc228212259)

[Figure S7 Subgroup analysis for OS (Low vs High LAR) in the internal validation set.	22](#_Toc228212260)

[Figure S8 Subgroup analysis for PFS (Low vs High LAR) in the internal validation set.	23](#_Toc228212261)

[Figure S9 Subgroup analysis for OS (Low vs High LAR) in the external test set.	24](#_Toc228212262)

[Figure S10 Subgroup analysis for PFS (Low vs High LAR) in the external test set.	25](#_Toc228212263)

[Figure S11 DCA of OS and PFS prediction by the nomograms.	26](#_Toc228212264)

[Figure S12 Online dynamic nomograms for OS and PFS.	27](#_Toc228212265)

# Supplementary Tables

## Table S1 Results from receiver operating characteristic analysis.

| Variables | Cut-off value | Sensitivity | Specificity | Positive predictive value | Negative  Predictive value | Youden index |
| --- | --- | --- | --- | --- | --- | --- |
| ALB | 39.3 | 0.65455 | 0.51323 | 0.43902 | 0.71852 | 0.16777 |
| LDH | 204.5 | 0.63636 | 0.77778 | 0.62500 | 0.78610 | 0.41414 |
| LAR | 5.1 | 0.64545 | 0.80952 | 0.66355 | 0.79688 | 0.45498 |

Note. ALB: albumin; LDH: lactate dehydrogenase; LAR: lactate dehydrogenase to albumin ratio.

## Table S2 Correlations between LAR and clinicopathological characteristics of patients in training set.

| **Characteristics** | **High** | **Low** |
| --- | --- | --- |
| n | 188 | 111 |
| Sex, n (%) |  |  |
| Male | 167 (55.9%) | 97 (32.4%) |
| Female | 21 (7%) | 14 (4.7%) |
| Age, n (%) |  |  |
| ˂65 | 101 (33.8%) | 63 (21.1%) |
| ≥65 | 87 (29.1%) | 48 (16.1%) |
| Hypertension, n (%) |  |  |
| YES | 58 (19.4%) | 35 (11.7%) |
| NO | 130 (43.5%) | 76 (25.4%) |
| Smoking History, n (%) |  |  |
| YES | 109 (36.5%) | 55 (18.4%) |
| NO | 79 (26.4%) | 56 (18.7%) |
| TNM Stage, n (%) |  |  |
| III | 54 (18.1%) | 74 (24.7%) |
| IV | 134 (44.8%) | 37 (12.4%) |
| Radiation Therapy, n (%) |  |  |
| YES | 95 (31.8%) | 74 (24.7%) |
| NO | 93 (31.1%) | 37 (12.4%) |
| Lymph Node Metastasis, n (%) |  |  |
| YES | 175 (58.5%) | 100 (33.4%) |
| NO | 13 (4.3%) | 11 (3.7%) |
| Types of targeted agents, n (%) |  |  |
| Anlotinib | 15 (5%) | 11 (3.7%) |
| Apatinib | 0 (0%) | 3 (1%) |
| NO | 173 (57.9%) | 97 (32.4%) |
| Chemotherapy, n (%) |  |  |
| EP | 128 (42.8%) | 72 (24.1%) |
| EC | 52 (17.4%) | 35 (11.7%) |
| Other | 8 (2.7%) | 4 (1.3%) |
| Immunotherapy, n (%) |  |  |
| Pembrolizumab | 5 (1.7%) | 3 (1%) |
| Durvalumab | 45 (15.1%) | 26 (8.7%) |
| Sintilimab | 27 (9%) | 25 (8.4%) |
| Serplulimab | 18 (6%) | 20 (6.7%) |
| Tislelizumab | 52 (17.4%) | 14 (4.7%) |
| Atezolizumab | 17 (5.7%) | 11 (3.7%) |
| Camrelizumab | 9 (3%) | 5 (1.7%) |
| Toripalimab | 6 (2%) | 2 (0.7%) |
| Other | 9 (3%) | 5 (1.7%) |
| Type of ICIs, n (%) |  |  |
| PD-1 | 122 (40.8%) | 71 (23.7%) |
| PD-L1 | 66 (22.1%) | 40 (13.4%) |
| Liver metastasis, n (%) |  |  |
| YES | 36 (12%) | 15 (5%) |
| NO | 152 (50.8%) | 96 (32.1%) |
| Bone metastasis, n (%) |  |  |
| YES | 46 (15.4%) | 11 (3.7%) |
| NO | 142 (47.5%) | 100 (33.4%) |
| Brain metastasis, n (%) |  |  |
| YES | 34 (11.4%) | 16 (5.4%) |
| NO | 154 (51.5%) | 95 (31.8%) |
| ECOG PS, n (%) |  |  |
| 0-1 | 132 (44.1%) | 96 (32.1%) |
| ≥2 | 56 (18.7%) | 15 (5%) |
| BMI, n (%) |  |  |
| <25 | 132 (44.1%) | 84 (28.1%) |
| ≥25 | 56 (18.7%) | 27 (9%) |
| LAR-continue, median (IQR) | 6.5912 (5.637, 8.151) | 4.4282 (4.027, 4.795) |
| CEA, median (IQR) | 4.835 (2.548, 14.100) | 3.4000 (2.263, 7.343) |
| NSE, median (IQR) | 36.200 (16.905, 83.532) | 25.655 (14.533, 57.163) |

Note. LAR: lactate dehydrogenase to albumin ratio; TNM: tumor–node–metastasis; ICIs: immune checkpoint inhibitors; ECOG PS: Eastern Cooperative Oncology Group Performance Status; BMI: Body Mass Index; CEA: carcinoembryonic antigen; NSE: neuron-specific enolase; IQR: Interquartile Range.

## Table S3 Correlations between LAR and clinicopathological characteristics of patients in internal validation set.

| **Characteristics** | **High** | **Low** |
| --- | --- | --- |
| n | 93 | 35 |
| Sex, n (%) |  |  |
| Male | 81 (63.3%) | 28 (21.9%) |
| Female | 12 (9.4%) | 7 (5.5%) |
| Age, n (%) |  |  |
| ˂65 | 54 (42.2%) | 21 (16.4%) |
| ≥65 | 39 (30.5%) | 14 (10.9%) |
| Hypertension, n (%) |  |  |
| YES | 24 (18.8%) | 7 (5.5%) |
| NO | 69 (53.9%) | 28 (21.9%) |
| Smoking History, n (%) |  |  |
| YES | 53 (41.4%) | 11 (8.6%) |
| NO | 40 (31.2%) | 24 (18.8%) |
| TNM Stage, n (%) |  |  |
| III | 33 (25.8%) | 18 (14.1%) |
| IV | 60 (46.9%) | 17 (13.3%) |
| Radiation Therapy, n (%) |  |  |
| YES | 40 (31.2%) | 24 (18.8%) |
| NO | 53 (41.4%) | 11 (8.6%) |
| Lymph Node Metastasis, n (%) |  |  |
| YES | 89 (69.5%) | 31 (24.2%) |
| NO | 4 (3.1%) | 4 (3.1%) |
| Types of targeted agents, n (%) |  |  |
| Anlotinib | 4 (3.1%) | 3 (2.3%) |
| Apatinib | 0 (0%) | 1 (0.8%) |
| NO | 89 (69.5%) | 31 (24.2%) |
| Chemotherapy, n (%) |  |  |
| EP | 61 (47.7%) | 26 (20.3%) |
| EC | 30 (23.4%) | 9 (7%) |
| Other | 2 (1.6%) | 0 (0%) |
| Immunotherapy, n (%) |  |  |
| Pembrolizumab | 1 (0.8%) | 1 (0.8%) |
| Durvalumab | 31 (24.2%) | 7 (5.5%) |
| Sintilimab | 8 (6.2%) | 9 (7%) |
| Serplulimab | 14 (10.9%) | 2 (1.6%) |
| Tislelizumab | 19 (14.8%) | 7 (5.5%) |
| Atezolizumab | 11 (8.6%) | 4 (3.1%) |
| Camrelizumab | 5 (3.9%) | 1 (0.8%) |
| Toripalimab | 1 (0.8%) | 1 (0.8%) |
| Other | 3 (2.3%) | 3 (2.3%) |
| Type of ICIs, n (%) |  |  |
| PD-1 | 49 (38.3%) | 22 (17.2%) |
| PD-L1 | 44 (34.4%) | 13 (10.2%) |
| Liver metastasis, n (%) |  |  |
| YES | 22 (17.2%) | 4 (3.1%) |
| NO | 71 (55.5%) | 31 (24.2%) |
| Bone metastasis, n (%) |  |  |
| YES | 22 (17.2%) | 6 (4.7%) |
| NO | 71 (55.5%) | 29 (22.7%) |
| Brain metastasis, n (%) |  |  |
| YES | 13 (10.2%) | 7 (5.5%) |
| NO | 80 (62.5%) | 28 (21.9%) |
| ECOG PS, n (%) |  |  |
| 0-1 | 67 (52.3%) | 32 (25%) |
| ≥2 | 26 (20.3%) | 3 (2.3%) |
| BMI, n (%) |  |  |
| ˂25 | 72 (56.2%) | 23 (18%) |
| ≥25 | 21 (16.4%) | 12 (9.4%) |
| LAR-continue, median (IQR) | 6.6321 (5.935, 8.545) | 4.121 (3.863, 4.590) |
| CEA, median (IQR) | 4.470 (2.398, 11.377) | 2.945 (1.978, 6.863) |
| NSE, median (IQR) | 37.330 (20.680, 69.735) | 23.455 (17.907, 42.760) |
|  |  |  |

Note. LAR: lactate dehydrogenase to albumin ratio; TNM: tumor–node–metastasis; ICIs: immune checkpoint inhibitors; PD-1: programmed cell death protein 1; PD-L1: programmed cell death ligand 1; ECOG PS: Eastern Cooperative Oncology Group Performance Status; BMI: Body Mass Index; CEA: carcinoembryonic antigen; NSE: neuron-specific enolase; IQR: Interquartile Range.

## Table S4 Correlations between LAR and clinicopathological characteristics of patients in external test set.

| **Characteristics** | **High** | **Low** |
| --- | --- | --- |
| n | 97 | 104 |
| Sex, n (%) |  |  |
| Male | 68 (33.8%) | 73 (36.3%) |
| Female | 29 (14.4%) | 31 (15.4%) |
| Age, n (%) |  |  |
| ˂65 | 58 (28.9%) | 67 (33.3%) |
| ≥65 | 39 (19.4%) | 37 (18.4%) |
| Hypertension, n (%) |  |  |
| YES | 43 (21.4%) | 44 (21.9%) |
| NO | 54 (26.7%) | 60 (29.9%) |
| Smoking History, n (%) |  |  |
| YES | 35 (17.4%) | 38 (18.9%) |
| NO | 62 (30.8%) | 66 (32.8%) |
| TNM Stage, n (%) |  |  |
| III | 68 (33.8%) | 76 (37.8%) |
| IV | 29 (14.4%) | 28 (13.9%) |
| Radiation Therapy, n (%) |  |  |
| YES | 77 (38.3%) | 93 (46.3%) |
| NO | 20 (10%) | 11 (5.5%) |
| Lymph Node Metastasis, n (%) |  |  |
| YES | 68 (33.8%) | 68 (33.8%) |
| NO | 29 (14.4%) | 36 (17.9%) |
| Types of targeted agents, n (%) |  |  |
| Anlotinib | 1 (0.5%) | 2 (1%) |
| Apatinib | 0 (0%) | 2 (1%) |
| NO | 96 (47.8%) | 100 (49.8%) |
| Chemotherapy, n (%) |  |  |
| EP | 91 (45.3%) | 98 (48.8%) |
| EC | 6 (3%) | 6 (3%) |
| Immunotherapy, n (%) |  |  |
| Durvalumab | 9 (4.5%) | 9 (4.5%) |
| Sintilimab | 64 (31.8%) | 67 (33.3%) |
| Serplulimab | 1 (0.5%) | 2 (1%) |
| Tislelizumab | 18 (9%) | 18 (9%) |
| Camrelizumab | 2 (1%) | 4 (2%) |
| Other | 3 (1.5%) | 4 (2%) |
| Type of ICIs, n (%) |  |  |
| PD-1 | 86 (42.8%) | 94 (46.8%) |
| PD-L1 | 11 (5.5%) | 10 (5%) |
| Liver metastasis, n (%) |  |  |
| YES | 14 (7%) | 20 (10%) |
| NO | 83 (41.3%) | 84 (41.8%) |
| Bone metastasis, n (%) |  |  |
| YES | 31 (15.4%) | 29 (14.4%) |
| NO | 66 (32.8%) | 75 (37.3%) |
| Brain metastasis, n (%) |  |  |
| YES | 19 (9.5%) | 24 (11.9%) |
| NO | 78 (38.8%) | 80 (39.8%) |
| ECOG PS, n (%) |  |  |
| 0-1 | 90 (44.8%) | 95 (47.3%) |
| ≥2 | 7 (3.5%) | 9 (4.5%) |
| BMI, n (%) |  |  |
| ˂25 | 56 (27.9%) | 52 (25.9%) |
| ≥25 | 41 (20.4%) | 52 (25.9%) |
| LAR-continue, median (IQR) | 6.1247 (5.3654, 7.3257) | 4.0395 (3.749, 4.4933) |

Note. LAR: lactate dehydrogenase to albumin ratio; TNM: tumor–node–metastasis; ICIs: immune checkpoint inhibitors; PD-1: programmed cell death protein 1; PD-L1: programmed cell death ligand 1; ECOG PS: Eastern Cooperative Oncology Group Performance Status; BMI: Body Mass Index; CEA: carcinoembryonic antigen; NSE: neuron-specific enolase; IQR: Interquartile Range.

## Table S5 Summary of confirmed response assessed by RECIST Version 1.1 in all patients.

| Summary of confirmed response assessed by RECIST Version 1.1 in training set. | | | |
| --- | --- | --- | --- |
| **Tumor Response** | **Low LAR** | **High LAR** | **P Value** |
| PD | 10（9%） | 45（23.9%） |  |
| PR | 46（41.4%） | 72（38.3%） |  |
| SD | 49（41.4%） | 66（34.0%） |  |
| Not Evaluable | 6（5.4%） | 5（2.7%） |  |
| ORR | 46（41.4%） | 72（38.3%） | 0.632 |
| DCR | 95（85.6%） | 138（73.4%） | 0.008 |
| Summary of confirmed response assessed by RECIST Version 1.1 in validation set. | | | |
| PD | 7(20.0%) | 23(24.7%) |  |
| PR | 11(31.4%) | 29(31.2%) |  |
| SD | 16(45.7%) | 36(38.7%) |  |
| Not Evaluable | 1(2.9%) | 5(5.3%) |  |
| ORR | 11(31.4%) | 29(31.2%) | 0.997 |
| DCR | 27(77.1%) | 65(69.9%) | 0.397 |

Note. RECIST: Response Evaluation Criteria In Solid Tumors; LAR: lactate dehydrogenase to albumin ratio; Objective response rate(ORR)= Complete response(CR) + Partial response(PR); Disease control rate (DCR)=Complete response (CR)+ Partial response(PR) + Stable disease (SD); Not evaluable = Patients who did not have one postbaseline imaging assessment.

| Table S6 Proportional hazards assumption test for the multivariable Cox regression model of OS and PFS in the training set | | | |
| --- | --- | --- | --- |
| Proportional hazards assumption test for the multivariable Cox regression model of OS | | | |
| **Characteristics** | Training set | |  |
|  | χ² value | P value |  |
| TNM Stage | 0.9199 | 0.3375 |  |
| ECOG PS | 0.8792 | 0.3484 |  |
| LAR | 2.1908 | 0.1388 |  |
| Global test | 3.9900 | 0.2625 |  |
| Proportional hazards assumption test for the multivariable Cox regression model of PFS | | | |
| **Characteristics** | Training set | |  |
| TNM Stage | 0.3298 | 0.5658 |  |
| ECOG PS | 0.1187 | 0.7305 |  |
| LAR | 0.1145 | 0.7350 |  |
| Global test | 0.5630 | 0.9049 |  |

Note. TNM: tumor–node–metastasis; ECOG PS: Eastern Cooperative Oncology Group Performance Status; LAR: lactate dehydrogenase to albumin ratio.

| Table S7 Comparison of C-index values for different prognostic models | | | | | |
| --- | --- | --- | --- | --- | --- |
| Comparison of C-index values for different prognostic models of OS | | | | |  |
| Model | Training set | Internal Validation set | External Test set |  |  |
| Nomogram with LAR | 0.750 (0.732–0.766) | 0.730 (0.700–0.754) | 0.652 (0.601, 0.703) | |  |
| Nomogram without LAR | 0.670 (0.651–0.689) | 0.660 (0.624–0.684) | 0.492 (0.439, 0.545) | |  |
| Comparison of C-index values for different prognostic models of PFS | | | | |  |
| Nomogram with LAR | 0.690 (0.667–0.704) | 0.690 (0.665–0.722) | 0.570 (0.524, 0.615) | |  |
| Nomogram without LAR | 0.640 (0.616–0.653) | 0.660 (0.630–0.685) | 0.500 (0.461, 0.540) | |  |

Note. LAR: lactate dehydrogenase to albumin ratio.

## Table S8 Results of univariate survival analysis in training, internal validation and external test sets.

| Univariate COX Regression analysis for OS | | | | |  |  |
| --- | --- | --- | --- | --- | --- | --- |
| Characteristics | **Training Set** | | **Internal Validation Set** | | **External Test Set** | |
|  | **Hazard ratio (95% CI)** | **P value** | **Hazard ratio (95% CI)** | **P value** | **Hazard ratio (95% CI)** | **P value** |
| Sex (Female vs Male) | 1.006 (0.649 - 1.560) | 0.978 | 0.986 (0.557 - 1.747) | 0.962 | 0.642 (0.412 - 0.999) | **0.050** |
| Age (≥65 vs ˂65) | 1.180 (0.886 - 1.573) | 0.257 | 0.996 (0.653 - 1.519) | 0.984 | 1.091 (0.739 - 1.609) | 0.662 |
| Hypertension (NO vs YES) | 1.067 (0.784 - 1.452) | 0.679 | 1.278 (0.782 - 2.088) | 0.328 | 1.040 (0.708 - 1.529) | 0.840 |
| Smoking History (NO vs YES) | 1.002 (0.753 - 1.334) | 0.989 | 0.918 (0.609 - 1.386) | 0.685 | 0.729 (0.485 - 1.095) | 0.128 |
| TNM Stage (IV vs III) | 2.699 (1.973 - 3.691) | **< 0.001** | 1.735 (1.129 - 2.667) | **0.012** | 0.929 (0.611 - 1.412) | 0.730 |
| Radiation Therapy (NO vs YES) | 1.451 (1.086 - 1.938) | 0.012 | 1.471 (0.964 - 2.244) | 0.074 | 1.628 (1.016 - 2.608) | 0.043 |
| Lymph Node Metastasis (NO vs YES) | 0.617 (0.344 - 1.109) | 0.106 | 0.380 (0.119 - 1.206) | 0.100 | 0.900 (0.596 - 1.359) | 0.617 |
| Types of targeted agents (Apatinib vs Anlotinib) | 1.087 (0.325 - 3.635) | 0.892 | 0.836 (0.100 - 6.975) | 0.868 | 0.591 (0.054 - 6.528) | 0.668 |
| Types of targeted agents (NO vs Anlotinib) | 1.199 (0.766 - 1.877) | 0.426 | 1.552 (0.675 - 3.569) | 0.301 | 1.068 (0.263 - 4.332) | 0.927 |
| Chemotherapy (EC vs EP) | 0.751 (0.540 - 1.044) | 0.088 | 0.766 (0.481 - 1.218) | 0.260 | 0.868 (0.380 - 1.983) | 0.738 |
| Chemotherapy (Other vs EP) | 1.305 (0.684 - 2.488) | 0.420 | 1.650 (0.398 - 6.830) | 0.490 | NA | NA |
| Type of ICIs (PD-L1 vs PD-1) | 0.822 (0.613 - 1.102) | 0.190 | 0.668 (0.440 - 1.014) | 0.058 | 0.935 (0.500 - 1.749) | 0.833 |
| Liver metastasis (NO vs YES) | 0.980 (0.684 - 1.405) | 0.912 | 0.937 (0.550 - 1.597) | 0.811 | 1.241 (0.738 - 2.089) | 0.416 |
| Bone metastasis (NO vs YES) | 0.671 (0.481 - 0.937) | **0.019** | 1.011 (0.609 - 1.678) | 0.967 | 0.981 (0.647 - 1.487) | 0.927 |
| Brain metastasis (NO vs YES) | 1.008 (0.691 - 1.469) | 0.968 | 1.807 (0.982 - 3.325) | 0.057 | 0.930 (0.585 - 1.479) | 0.760 |
| ECOG PS (≥2 vs 0-1) | 2.296 (1.671 - 3.154) | **< 0.001** | 3.534 (2.154 - 5.799) | **< 0.001** | 1.329 (0.670 - 2.634) | 0.416 |
| BMI (≥25 vs ˂25) | 1.318 (0.959 - 1.811) | 0.088 | 0.809 (0.507 - 1.291) | 0.374 | 0.722 (0.490 - 1.062) | 0.098 |
| LAR (High vs Low) | 1.022 (1.010 - 1.034) | **< 0.001** | 5.116 (2.833 - 9.240) | **< 0.001** | 1.203 (1.103 - 1.313) | **< 0.001** |
| CEA | 1.000 (1.000 - 1.001) | 0.246 | 1.002 (1.000 - 1.004) | 0.102 | NA | NA |
| NSE | 1.001 (0.999 - 1.003) | 0.333 | 1.003 (1.000 - 1.006) | 0.061 | NA | NA |
| Univariate COX Regression analysis for PFS | | | | | | |
| Sex (Female vs Male) | 1.375 (0.937 - 2.020) | 0.104 | 1.199 (0.722 - 1.991) | 0.483 | 0.921 (0.660 – 1.286) | 0.629 |
| Age (≥65 vs ˂65) | 0.828 (0.640 - 1.072) | 0.152 | 0.858 (0.590 - 1.248) | 0.422 | 1.012 (0.747 – 1.371) | 0.937 |
| Hypertension (NO vs YES) | 1.035 (0.787 - 1.362) | 0.804 | 0.926 (0.604 - 1.421) | 0.725 | 1.093 (0.809 – 1.476) | 0.563 |
| Smoking History (NO vs YES) | 0.925 (0.715 - 1.197) | 0.553 | 1.192 (0.827 - 1.718) | 0.347 | 1.034 (0.757 – 1.413) | 0.832 |
| TNM Stage (IV vs III) | 2.310 (1.755 - 3.040) | **< 0.001** | 1.529 (1.052 - 2.223) | **0.026** | 0.843 (0.606 – 1.171) | 0.308 |
| Radiation Therapy (NO vs YES) | 1.331 (1.029 - 1.723) | **0.029** | 1.830 (1.237 - 2.708) | **0.002** | 1.029 (0.686 – 1.543) | 0.891 |
| Lymph Node Metastasis ((NO vs YES) | 0.757 (0.455 - 1.260) | 0.285 | 0.524 (0.228 - 1.207) | 0.129 | 0.903 (0.656 – 1.243) | 0.532 |
| Types of targeted agents (Apatinib vs Anlotinib) | 1.075 (0.323 - 3.577) | 0.906 | 0.576 (0.266 - 1.244) | 0.160 | 3.229 (0.532 – 19.613) | 0.203 |
| Types of targeted agents (NO vs Anlotinib) | 1.027 (0.673 - 1.569) | 0.900 | 0.294 (0.040 - 2.138) | 0.227 | 1.259 (0.401 – 3.954) | 0.693 |
| Chemotherapy (EC vs EP) | 0.837 (0.630 - 1.113) | 0.222 | 1.000 (0.670 - 1.493) | 0.998 | 0.995 (0.539 – 1.837) | 0.986 |
| Chemotherapy (Other vs EP) | 1.130 (0.594 - 2.149) | 0.709 | 2.466 (0.597 - 10.177) | 0.212 | NA | NA |
| Type of ICIs (PD-L1 vs PD-1) | 0.978 (0.753 - 1.270) | 0.868 | 1.092 (0.750 - 1.590) | 0.646 | 0.707 (0.422 – 1.185) | 0.188 |
| Liver metastasis (NO vs YES) | 0.938 (0.676 - 1.303) | 0.703 | 1.280 (0.790 - 2.073) | 0.316 | 1.223 (0.810 – 1.847) | 0.337 |
| Bone metastasis (NO vs YES) | 0.841 (0.611 - 1.159) | 0.290 | 0.814 (0.515 - 1.286) | 0.378 | 1.140 (0.818 – 1.590) | 0.439 |
| Brain metastasis (NO vs YES) | 0.919 (0.652 - 1.296) | 0.631 | 0.828 (0.500 - 1.372) | 0.464 | 0.766 (0.534 – 1.098) | 0.147 |
| ECOG PS (≥2 vs 0-1) | 2.224 (1.659 - 2.981) | **< 0.001** | 3.941 (2.530 - 6.140) | **< 0.00**1 | 1.688 (1.007 – 2.831) | 0.047 |
| BMI (≥25 vs ˂25) | 1.202 (0.904 - 1.598) | 0.205 | 0.755 (0.498 - 1.146) | 0.187 | 1.253 (0.930 – 1.688) | 0.137 |
| LAR (High vs Low) | 1.018 (1.009 - 1.027) | **< 0.001** | 3.660 (2.190 - 6.117) | **< 0.001** | 1.056 (0.979 – 1.139) | 0.155 |
| CEA | 1.000 (1.000 - 1.001) | 0.758 | 1.003 (1.001 - 1.005) | **0.015** | NA | NA |
| NSE | 1.001 (0.999 - 1.002) | 0.456 | 1.000 (0.997 - 1.003) | 0.895 | NA | NA |

**Note.** TNM, tumor–node–metastasis; EP, Etoposide+Cisplatin; EC, Etoposide+Carboplatin; ICIs, immune checkpoint inhibitors; PD-1, programmed cell death protein 1; PD-L1, programmed cell death ligand 1; ECOG PS, Eastern Cooperative Oncology Group Performance Status; LAR, lactate dehydrogenase to albumin ratio; BMI, body mass index; CEA, carcinoembryonic antigen; NSE, neuron-specific enolase. OS, overall survival; PFS: progression-free survival; CI: Confidence Interval.

## Table S9 Results of multivariable Cox regression analysis in training, internal validation and external test sets.

| Multivariable COX Regression analysis for OS | | | | |  |  |
| --- | --- | --- | --- | --- | --- | --- |
| Characteristics | **Training Set** | | **Internal Validation Set** | | **External Test Set** | |
|  | **Hazard ratio (95% CI)** | **P value** | **Hazard ratio (95% CI)** | **P value** | **Hazard ratio (95% CI)** | **P value** |
| Sex (Female vs Male) |  |  |  |  | 0.700 (0.447 - 1.096) | 0.119 |
| Age (≥65 vs ˂65) |  |  |  |  |  |  |
| Hypertension (NO vs YES) |  |  |  |  |  |  |
| Smoking History (NO vs YES) |  |  |  |  |  |  |
| TNM Stage (IV vs III) | 2.451 (1.770 - 3.394) | **< 0.001** | 1.604 (1.005 - 2.562) | **0.048** |  |  |
| Radiation Therapy (NO vs YES) | 1.210 (0.902 - 1.623) | 0.204 |  |  |  |  |
| Lymph Node Metastasis (NO vs YES) |  |  |  |  |  |  |
| Types of targeted agents (Apatinib vs Anlotinib) |  |  |  |  |  |  |
| Types of targeted agents (NO vs Anlotinib) |  |  |  |  |  |  |
| Chemotherapy (EC vs EP) |  |  |  |  |  |  |
| Chemotherapy (Other vs EP) |  |  |  |  |  |  |
| Type of ICIs (PD-L1 vs PD-1) |  |  |  |  |  |  |
| Liver metastasis (NO vs YES) |  |  |  |  |  |  |
| Bone metastasis (NO vs YES) | 0.831 (0.587 - 1.177) | 0.297 |  |  |  |  |
| Brain metastasis (NO vs YES) |  |  | 1.462 (0.778 - 2.746) | 0.238 |  |  |
| ECOG PS (≥2 vs 0-1) | 2.259 (1.637 - 3.118) | **< 0.001** | 2.777 (1.634 - 4.720) | **< 0.001** |  |  |
| BMI (≥25 vs ˂25) |  |  |  |  |  |  |
| LAR-continue | 1.022 (1.007 - 1.038) | **0.005** | 5.066 (2.736 - 9.379) | **< 0.001** | 1.189 (1.090 - 1.298) | **< 0.001** |
| CEA |  |  |  |  |  |  |
| NSE |  |  |  |  |  |  |
| Multivariable COX Regression analysis for PFS | | | | | | |
| Sex (Female vs Male) |  |  |  |  |  |  |
| Age (≥65 vs ˂65) |  |  |  |  |  |  |
| Hypertension (NO vs YES) |  |  |  |  |  |  |
| Smoking History (NO vs YES) |  |  |  |  |  |  |
| TNM Stage (IV vs III) | 2.357 (1.785 - 3.112) | **< 0.001** | 0.994 (0.660 - 1.497) | 0.976 |  |  |
| Radiation Therapy (NO vs YES) | 1.195 (0.923 - 1.549) | 0.177 | 1.356 (0.886 - 2.075) | 0.161 |  |  |
| Lymph Node Metastasis ((NO vs YES) |  |  |  |  |  |  |
| Types of targeted agents (Apatinib vs Anlotinib) |  |  |  |  |  |  |
| Types of targeted agents (NO vs Anlotinib) |  |  |  |  |  |  |
| Chemotherapy (EC vs EP) |  |  |  |  |  |  |
| Chemotherapy (Other vs EP) |  |  |  |  |  |  |
| Type of ICIs (PD-L1 vs PD-1) |  |  |  |  |  |  |
| Liver metastasis (NO vs YES) |  |  |  |  |  |  |
| Bone metastasis (NO vs YES) |  |  |  |  |  |  |
| Brain metastasis (NO vs YES) |  |  |  |  |  |  |
| ECOG PS (≥2 vs 0-1) | 2.263 (1.685 - 3.040) | **< 0.001** | 4.515 (2.754 - 7.403) | **< 0.001** |  |  |
| BMI (≥25 vs ˂25) |  |  |  |  |  |  |
| LAR-continue | 1.021 (1.011 - 1.032) | **< 0.001** | 3.204 (1.851 - 5.545) | **< 0.001** |  |  |
| CEA |  |  | 1.002 (0.999 - 1.004) | 0.151 |  |  |
| NSE |  |  |  |  |  |  |

**Note.** TNM, tumor–node–metastasis; EP, Etoposide+Cisplatin; EC, Etoposide+Carboplatin; ICIs, immune checkpoint inhibitors; PD-1, programmed cell death protein 1; PD-L1, programmed cell death ligand 1; ECOG PS, Eastern Cooperative Oncology Group Performance Status; LAR, lactate dehydrogenase to albumin ratio; BMI, body mass index; CEA, carcinoembryonic antigen; NSE, neuron-specific enolase. OS, overall survival; PFS: progression-free survival; CI: Confidence Interval.

# Supplementary Figures

## Figure S1 The ROC curves of training set

.

## Figure S2 Kaplan–Meier curves for OS and PFS stratified by the LAR in the training set and internal validation set.

Kaplan–Meier curves for OS and PFS of patients in stage III (A, B) and IV (E, F) in the training set, Kaplan–Meier curves for OS and PFS of patients in stage III (C, D) and IV (G, H) in the validation set. LAR: lactate dehydrogenase to albumin ratio; HR: Hazard Ratio; OS: overall survival; PFS: Progression-Free Survival.

## Figure S3 Risk Factor Map of training set.

PFS: progression-free survival; NSE: neuron-specific enolase; CEA: carcinoembryonic antigen; LAR: lactate dehydrogenase to albumin ratio; ECOG PS: Eastern Cooperative Oncology Group Performance Status; TNM: tumor–node–metastasis; ICIs: immune checkpoint inhibitors; BMI: Body Mass Index; PD-1: programmed cell death protein 1; PD-L1: programmed cell death ligand 1.

## Figure S4 Risk Factor Map of validation set.

PFS: progression-free survival; NSE: neuron-specific enolase; CEA: carcinoembryonic antigen; LAR: lactate dehydrogenase to albumin ratio; ECOG PS: Eastern Cooperative Oncology Group Performance Status; TNM: tumor–node–metastasis; ICIs: immune checkpoint inhibitors; BMI: Body Mass Index; PD-1: programmed cell death protein 1; PD-L1: programmed cell death ligand 1.

## Figure S5 Subgroup analysis for OS (Low vs High LAR) in the training set.

LAR: lactate dehydrogenase to albumin ratio; HR: Hazard Ratio; CI: Confidence Interval; TNM: tumor–node–metastasis; ICIs: immune checkpoint inhibitors; PD-1: programmed cell death protein 1; PD-L1: programmed cell death ligand 1; ECOG PS: Eastern Cooperative Oncology Group Performance Status; BMI: Body Mass Index; OS: overall survival.

## Figure S6 Subgroup analysis for PFS (Low vs High LAR) in the training set.

LAR: lactate dehydrogenase to albumin ratio; HR: Hazard Ratio; CI: Confidence Interval; TNM: tumor–node–metastasis; ICIs: immune checkpoint inhibitors; PD-1: programmed cell death protein 1; PD-L1: programmed cell death ligand 1; ECOG PS: Eastern Cooperative Oncology Group Performance Status; BMI: Body Mass Index; PFS: progression-free survival.

## Figure S7 Subgroup analysis for OS (Low vs High LAR) in the internal validation set.

LAR: lactate dehydrogenase to albumin ratio; HR: Hazard Ratio; CI: Confidence Interval; TNM: tumor–node–metastasis; ICIs: immune checkpoint inhibitors; PD-1: programmed cell death protein 1; PD-L1: programmed cell death ligand 1; ECOG PS: Eastern Cooperative Oncology Group Performance Status; BMI: Body Mass Index; OS: overall survival.

## Figure S8 Subgroup analysis for PFS (Low vs High LAR) in the internal validation set.

LAR: lactate dehydrogenase to albumin ratio; HR: Hazard Ratio; CI: Confidence Interval; TNM: tumor–node–metastasis; ICIs: immune checkpoint inhibitors; PD-1: programmed cell death protein 1; PD-L1: programmed cell death ligand 1; ECOG PS: Eastern Cooperative Oncology Group Performance Status; BMI: Body Mass Index; PFS: progression-free survival.

## Figure S9 Subgroup analysis for OS (Low vs High LAR) in the external test set.

LAR: lactate dehydrogenase to albumin ratio; HR: Hazard Ratio; CI: Confidence Interval; TNM: tumor–node–metastasis; ICIs: immune checkpoint inhibitors; PD-1: programmed cell death protein 1; PD-L1: programmed cell death ligand 1; ECOG PS: Eastern Cooperative Oncology Group Performance Status; BMI: Body Mass Index; PFS: progression-free survival.

## Figure S10 Subgroup analysis for PFS (Low vs High LAR) in the external test set.

LAR: lactate dehydrogenase to albumin ratio; HR: Hazard Ratio; CI: Confidence Interval; TNM: tumor–node–metastasis; ICIs: immune checkpoint inhibitors; PD-1: programmed cell death protein 1; PD-L1: programmed cell death ligand 1; ECOG PS: Eastern Cooperative Oncology Group Performance Status; BMI: Body Mass Index; PFS: progression-free survival.

## Figure S11 DCA of OS and PFS prediction by the nomograms.

The DCA of the nomogram for 1-year OS (A), 2-year OS (B), and for 1-year PFS (C), 2-year PFS (D) in the training set. DCA of the nomogram for 1-year OS (E), 2-year OS (F) and for 1-year PFS (G), 2-year PFS (H) in the internal validation set. DCA of the nomogram for 1-year OS (I), 2-year OS (J) and for 1-year PFS (K), 2-year PFS (L) in the external test set. DCA: decision curve analysis; OS: overall survival; PFS: progression-free survival.

## Figure S12 Online dynamic nomograms for OS and PFS.

Online dynamic nomograms accessible at [https://lar-clinical-os.shinyapps.io/dynnomapp/](https://lar-clinical.shinyapps.io/dynnomapp/) for OS (A); online dynamic nomograms accessible at <https://lar-clinical-pfs.shinyapps.io/dynnomapp/> for PFS (B). OS: overall survival; PFS: progression-free survival.
